# Atorvastatin attenuates the ovarian damage induced by cyclophosphamide in rat: An experimental study

**Published:** 2018-05

**Authors:** Maedeh Hamzeh, Seyed Jalal Hosseinimehr, Hamid Reza Mohammadi, Saeed Yaghubi Beklar, Ayat Dashti, Fereshteh Talebpour Amiri

**Affiliations:** 1 *Department of Anatomy, Faculty of Medicine, Molecular and Cell Biology Research Center, Mazandaran University of Medical Sciences, Sari, Iran*; 2 *Student Research Committee, Faculty of Medicine, Mazandaran University of Medical Sciences, Sari, Iran*; 3 *Department of Radiopharmacy, Faculty of Pharmacy, Mazandaran University of Medical Sciences, Sari, Iran *; 4 *Department of Toxicology and Pharmacology, Faculty of Pharmacy, Mazandaran University of Medical Sciences, Sari, Iran*

**Keywords:** Cyclophosphamide, Atorvastatin, Ovary, Oxidative stress, Antioxidant

## Abstract

**Background:**

Cyclophosphamide (CP), as an anticancer agent, causes ovarian toxicity and subsequent infertility in women. Atorvastatin (ATV) at a low dose has antioxidant and anti-inflammatory properties.

**Objective:**

The aim of this study was to investigate the protective effect of ATV against CP-induced ovarian injury in rat.

**Materials and Methods:**

In this experimental study, thirty-two female Wistar rats were randomly divided into four groups as I) control, II) ATV (10 mg/kg), III) CP (150 mg/kg), and IV) CP +ATV. The ATV treated groups were received ATV for 10 days via oral gavage. In the CP+ATV group, ATV was administrated on 5 days before and 5 days after CP injection. Histological structure, apoptosis (caspase-3), oxidative stress parameters as malondialdehyde, reactive oxygen species, protein carbonyl levels and cell viability were evaluated in ovary tissue by histological scores, immunohistochemistry, histochemical and biochemical assays. The levels of estrogen and progesterone hormones were measured on the 12th day of study.

**Results:**

ATV pretreatment significantly decreased the levels of oxidative stress biomarkers as malondialdehyde, reactive oxygen species and protein carbonyl levels and increased cell death in CP-treated rats as compared with the CP alone group. ATV significantly increased estrogen and progesterone levels in CP-treated rats. In addition, the histological examination showed ATV mitigated acute inflammation, degenerative cells in stroma and follicles, stromal edema, vacuolization, atresia of the follicles and congestion of blood vessels in the CP-treated animals. Furthermore, ATV significantly reduced immunoreactivity level of caspase-3 in CP-treated rats.

**Conclusion:**

Our results showed that the ATV with antioxidant and anti-apoptosis (caspase-3) activities protected ovarian against CP-induced toxicity.

## Introduction

Mortality in women with cancer decreased in recent decades due to advances in diagnosis, treatment and surgical techniques as well as increased public awareness to primary care and preventive strategies (1). Unfortunately, chemotherapy leads to side effects specially in young female patients such as reducing the number of primordial and other growing follicles which leading to early menopause and infertility (2). Cyclophosphamide (CP) is an effective alkylating anticancer agent that widely used in patients with cancer. Therapeutic and toxic effects of CP are related to its active metabolites as acrolein and phosphoramide mustard. These metabolites bind to DNA lead to disruption of DNA synthesis and cell death (3). CP metabolites produce reactive oxygen species (ROS) by conjugation with glutathione results in interference with the antioxidant defense system of the ovary (4). Malondialdehyde (MDA) as a marker of lipid peroxidation was increased following treatment to CP in the ovary of rats (5, 6). 

CP effects on the granulosa cell proliferation and secretion of ovarian hormones results in ovarian failure. The toxic effects of CP on the ovary include reducing the number of primordial, preantral, and antral follicles associated with decreased levels of progesterone and estradiol hormones (7). The number of primordial and growing follicles decreases in female of mice that were treated with CP, so that in their reproductive lifespan was decreased. The amenorrhea and infertility were other side effects of chemotherapy (7). CP is accompanied with a major risk of women temporary or permanent amenorrhea, reduced fertility, or infertility (8).

Atorvastatin (ATV) is an inhibitor of 3-hydroxy-3-methyl-glutarylcoenzyme A (HMG Co-A) reductase inhibits cholesterol that belongs to statin. Atorvastatin (ATV), at low doses has been prescribed for the treatment of hyperlipidemia. ATV addition to lowering cholesterol level, has therapeutic beneficial pleiotropic effects that happen at low dose (9). Biological and pharmacological properties of ATV are anti-inflammatory (10) antioxidant properties (11). On the other hand, high-dose atorvastatin is associated with many complications such as nephrotoxicity (12), testicular injury (13). But, atorvastatin at low dose does not have side effects on fertility and reproduction (14). Protective effect of atorvastatin was reported against nephrotoxicity (15), cardiotoxicity (16), testicular toxicity (17) and ovarian toxicity (18). So far, no study has not reported on the protective effect of ATV against ovarian injury induced by CP. 

The objective of our study was to investigate the effects of ATV on the prevention of ovarian damage induced by CP through histological, biochemical, hormonal and immunohistochemical assessments.

## Materials and methods

**Chemicals**

Atorvastatin was obtained from Sobhan Darou Pharmaceutical Company (Rasht, Iran). The progesterone and estrogen levels were measured by using Enzyme-linked Immunosorbent Assay Kit in accordance with the manufacturer’s protocol (Cat.No.E0243Mo and Cat.No.E0259Mo; China). Cyclophosphamide was purchased from Baxer Company (Germany). 

**Experimental animals**

Thirty-two female Wistar Albino rats (180-200 gr) were obtained from Animal Research Center of Mazandaran University of Medical Sciences, Sari, Iran. The animals were housed in suitable conditions of 12 hr light/ dark cycle, humidity (55±5%) and temperature (23±2^o^C). 

**Study design**

In experimental study, the rats were randomly divided to 4 groups (8 rats per each group): 

I; Rats were received phosphate buffered saline (same volume with other groups) as a vehicle of ATV for 10 consecutive days (no-treated CP or ATV).

II; Rats were administered 10 mg/kg ATV daily for 10 consecutive days with oral gavage without receiving CP (13).

III; Rats were administered CP single dose intraperitoneally at a dose 150 mg/kg on the 5 days (5).

IV; Rats were received 10 mg/kg ATV daily for 10 consecutive days and on the 5 day were received CP single dose.

**Figure 1:**
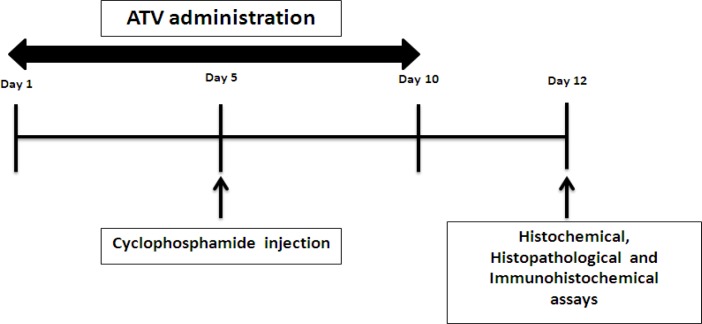
Study design diagram for evaluating the effect of atorvastatin (ATV) on ovarian damage induced cyclophosphamide (CP). ATV was prescribed for 10 consecutive days, rats were administered CP single dose intraperitoneally at a dose 150 mg/kg on day 5. Histochemical, histopathological and immunohistochemical assays were performed on day 12 of the study

The duration and dose of atorvastatin were based on previous studies (13). The most appropriate time for the assessment of histochemistry is 24-72 hr after receiving the latest medication. So, in this study, we evaluated the effect of atorvastatin on the histochemistry and histopathological structure of the ovary, two days after receiving the last medicine Figure 1.

**Mitochondrial preparation of ovarian tissues**

The mitochondria were prepared for the evaluation of oxidative stress markers from ovarian tissue. Mitochondria of ovaries were removed and minced in a cold mannitol (225 mM) solution and then homogenized by homogenizer. Homogenized tissue was centrifuged (1500×g, 10 min) at 4^o^C. Supernatant was removed for further centrifugation (11,000×g, 10 min) and then superior layer was discarded. The sedimented mitochondrial pellet was gently washed and then suspended in the isolation medium (0.225 M D-mannitol, 75 mM sucrose, and 0.2 mM EDTA, pH=7.4) and after that centrifuged again (11,000×g for 10 min). The resulting mitochondria were suspended in Tris buffer.A respiratory buffer (0.32 mM sucrose,10 mM Tris, 20 mM Mops, 50 μM EGTA, 0.5 mM MgCl_2_, 0.1 mM KH_2_PO_4_ and 5 mM sodium succinate) was used to measure the production of ROS. The ovarian tissue mitochondria were prepared within 4 hr of isolation. (19).

**Protein Concentration**

Concentrations of Mitochondrial protein were carried out using the method developed by Bradford. Briefly, 100 ml of suspensions of mitochondrial were added to each well of 96-well plate and then gently mixed with Bradford reagent. The absorbance of protein concentrations was measured at 595 nm (20).

**Biochemical analysis**

**ROS assay in mitochondria**

ROS levels of mitochondria as biomarkers of oxidative stress were measured using the Dichloro-dihydro-fluorescein diacetate reagent. Briefly, isolated ovarian mitochondria (0.5 mg mitochondrial protein/ml) were placed in respiration buffer and then 20 μL dichloro-dihydro-fluorescein diacetate was added to the samples added (final concentration, 10 μM) and incubated at 37^o^C for 15 min. Then absorption was measured by fluorescence spectrophotometer (Shimadzu RF5000U, Tokyo, Japan) at the λEx=488 nm and λEm=527 nm (21). 

**Evaluation of MDA formation in mitochondria**

The concentrations of MDA as lipid peroxidation were measured by estimating of MDA using the thiobarbituric acid with a spectrophotometric assay. To begin the analysis, to the 0.2 mL of sample, 0.25 mL phosphoric acid (0.05 M) was added and after that 0.3 mL of 0.2% thiobarbituric acid was added. Samples were reserved in a boiling water bath for 30 min. The sample tubes were placed to the ice-bath and then 0.4 mL of n-butanol was added to each. Samples were centrifuged (3500 rpm) for 10 min and MDA was measured based on reacts with thiobarbituric acid (an MDA- thiobarbituric acid complex). Created MDA in each sample was calculated in the supernatant at 532 nm with ELISA reader (Tecan, Rainbow Thermo, Austria). The content of formed MDA was expressed as nmol/mg protein. Tetramethoxypropane was used in this experiment as standard (19). 

**Evaluation of protein carbonyl content in mitochondria**

The protein carbonyl as protein peroxidation was measured by using 2, 4-dinitrophenyl-hydrazine (DNPH) reagent. After determination of tissue protein, 500 μL of trichloroacetic acid (20% w / v) was added to the sample and stored at 4^o^C for 15 min. Then precipitated protein was centrifuged at 6500×g for 10 min and the supernatant was discarded. Soluble protein (0.5 mL) was reacted with DNPH 10 mM (0.5 mL) in HC1 2 M for 1 hr at room temperature. precipitated was washed with 1 ml of a mixture of ethanol and ethyl acetate 1: 1 (v/v) and then centrifuged at 6500×g for 10 min and the supernatant was removed. The final protein (microtube contents) deposition solubilized in 200 μL Guanine hydrochloride solution and was centrifuged at 16,000×g for 5 min to remove any trace of insoluble material. The protein carbonyl was assessed spectrophotometrically by reading the absorption at a wavelength of 365 nm with an absorption coefficient of 22,000 M-1 cm-1 was expressed as a nmol of DNPH per milligram of protein (19). 

**Cell viability assay**

Mitochondrial function, as cell and mitochondria viability, was assessed using the MTT (3-[4, 5- dimethylthiazol-2-yl]-2, 5-diphenyltetrazolium bromide) assay based on reduction of MTT to its formazan product by mitochondrial dehydrogenase activity of ovarian tissue. In this way, after preparation of the samples, MTT dissolved in phosphate buffered saline (pH=7.2, at 2.5 mg/ml) and was added to the samples. After incubation at 37^o^C for 0.5 hr, mitochondrial toxicity was measured by assessing the reduction of MTT (3-[4, 5-dimethylthiazol-2-yl]-2, 5-diphenyltetrazolium bromide by mitochondria at 580 nm (22).

**Estrogen and progesterone assay**

Levels of estrogen and progesterone hormones in serum were determined by using spectrophotometry according to manufacturer's instructions (Mouse Estrogen ELISA Kit, Bioassay, cat. No. E0243Mo, China) and (Mouse progesterone ELISA Kit, Bioassay, Cat. No. E0259Mo, China). Estrogen and progesterone amount in the samples were calculated from standard curves using a linear regression method and expressed as ng/l. All samples were carried in duplicate. All spectrophotometric methods were performed by ELISA Reader (ELISA Microplate Readers, ELX800, BioTek Company) for hormonal and tissue biochemical evaluation.

**Histopathological examination**

The right ovary was fixed in 10% formalin buffer solution. After tissue processing, embedding in paraffin, tissue sections with a thickness of 5 μm were stained with hematoxylin and eosin. The change in tissue structure was graded from 0 to 4 in terms of severity of interstitial edema, vascular dilatation, hemorrhage, and neutrophil infiltrations (23). An Olympus light microscope (Olympus, Tokyo, Japan) was used for histological evaluation. The histological sections were performed by the histologist in a blinded fashion. 

**Immunohistochemical analysis**

For immunohistochemical assay using the kit, at first samples were deparaffined and rehydrated. Endogenous peroxidase activity of samples was blocked (0.3% H_2_O_2_ in methanol, 30 min). Then, samples were incubated with primary antibodies (4^o^C, overnight, anti-caspase-3 rabbit polyclonal antibody, 1:100 in phosphate buffered saline, v/v, Abcam, lat: GR224831-2). After incubation (2 hr) with secondary antibody conjugated with horseradish peroxidase (Mouse and Rabbit Specific HRP/DAB, Abcam, Lat: GR2623314-4), sections were incubated (5 min) with diaminobenzidine tetrahydrochloride. Then, the samples were dehydrated and mounted (17). 

The immunohistochemical photomicrographs were evaluated by using Mac Biophotonics ImageJ 1.41a software. The positive staining severity was assessed as the ratio of the stained area to the entire field assessment. Samples examined who was blinded to treatment.

**Ethical consideration**

Rats were fed standard pellet chow and had free access to water. Animals in each group were kept in polypropylene cages. All the experimental procedures were conducted by the institutional animal. All the experimental procedures were conducted by the Institutional Animal Ethics Committee of the Mazandaran University Medical Sciences (IR.MAZUMS.REC.1396.3039).

**Statistical analysis**

Statistical analysis of the obtained data was performed using SPSS (Statistical Package for the Social Sciences, version 19.0, SPSS Inc, Chicago, Illinois, USA). The normality of data was evaluated using the K-S (Kolmogorov-Smirnov) test, and then results with normally distributed was checked by One-Way ANOVA and followed by Tukey’s procedure. Data were expressed as the mean ± standard deviation (SD). Statistically significant differences were accepted as p<0.05.

## Results

**Effect of ATV on oxidative stress biomarkers in CP treated rats**

**Figure 2:**
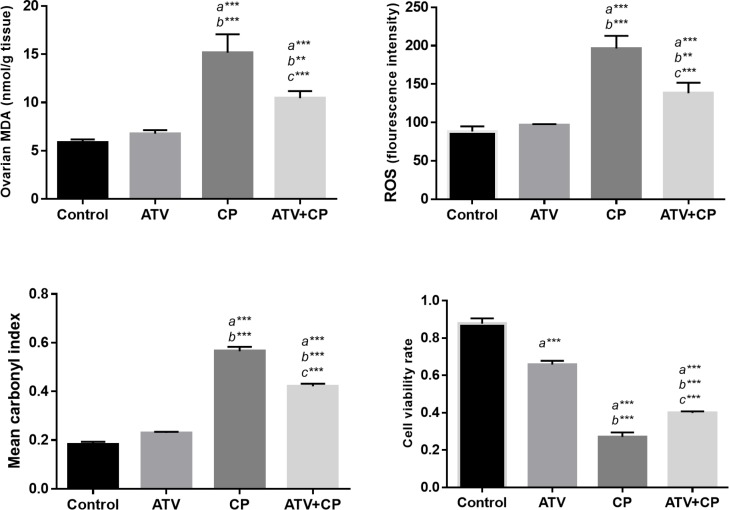
Histogram shows the levels of MDA, ROS, protein carbonyl and cell viability rate. All values are expressed as mean ± SD. a vs. the control; b vs. ATV; c vs. CP groups. **; p≤0.01, *** and p<0.001.

The MDA, ROS, PC levels and cell viability rate in ovary tissue are presented in Figure 2. Intracellular ROS formation significantly increased in CP-treated rats when compared with control group (p<0.001). Administration of ATV at the dose of 10 mg/kg significantly decreased the elevated ROS in ATV+CP group as compared to CP group (p<0.01). No significant alterations were observed in ROS levels in animals that were received only ATV as compared with control group. Ovarian MDA level in CP group was increased significantly (p<0.001) compared to control group. ATV treatment significantly inhibited elevation of lipid peroxidation (MDA levels) in ovary compared to CP-treated group (p<0.01). Moreover, CP induced clearly lipid peroxidation in the ovary by about 3-fold compared with the control group. The amount of MDA levels was same in the control and ATV groups.

**Figure 3:**
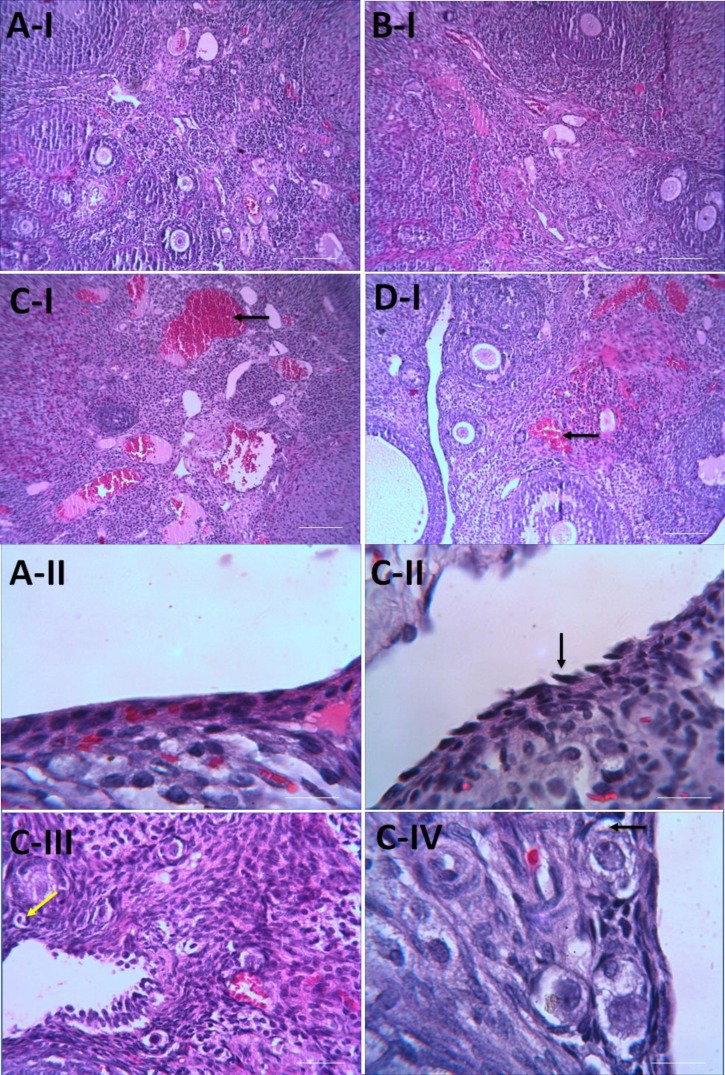
Photomicrographs of effect of ATV pre-treatment on the histological architecture of ovary in the groups. Normal structure in control (A) and ATV (B) groups, atretic follicle (C-III, yellow arrow) and congestion blood vessel in CP group (C-I, black arrow), detachment of germinal epithelium (C-II, black arrow) and vacuolization (C-IV, black arrow). Treatment with ATV almost improves these changes. (H & E staining. Mag: (A-I, B-I, C-I, D-I) ×10, (A-II, C-II, C-IV) ×100, (C-III) ×40. Scale bar=150 µm.

**Figure 4:**
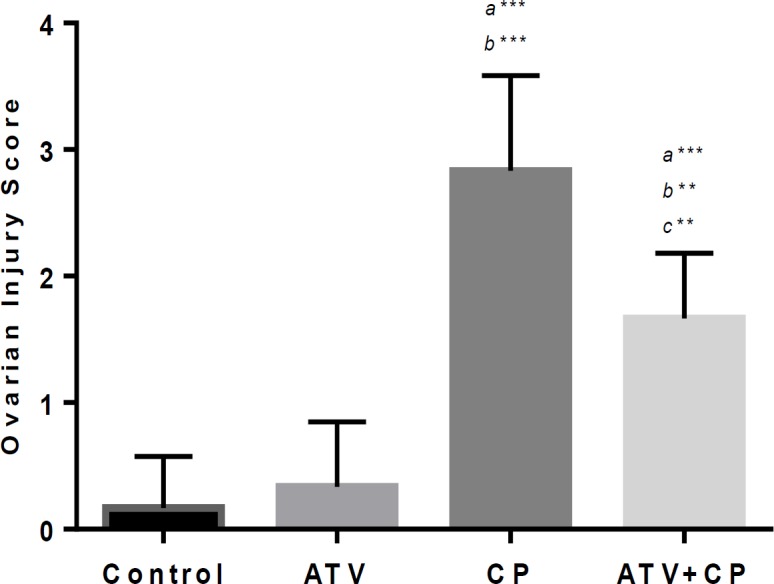
Histogram shows injury score in ovary tissue. Data are presented as Mean ± SD. a vs. the control; b vs. ATV; c vs. CP groups. **; p<0.01, *** and p<0.001.

Carbonyl contents, as an index for oxidative modification of proteins, were increased significantly in ovaries of CP-treated rat (p<0.001) as compared with control and ATV groups. ATV mitigated increasing protein carbonyl content in the ovarian tissue of CP-treated rat (p<0.001) compared with CP alone group. The amounts of PC were insignificant in the control and ATV groups (Figure 3). Cell viability was measured with MTT test. Decrease in cell viability and the most toxicity of ovarian tissue was seen in CP-treated rats (30.81%). Treatment of rats with ATV for 10 days in CP treated rats showed increased cell viability by 45.46%. ATV alone decreased cell viability rate in the ovaries, and this difference was statistically significant compared to the control group (75.01% vs. 100% at p<0.001). The results suggested that the combination of CP and ATV mitigate the cytotoxic effect of CP (Figure 4).

**Effect of ATV on estrogen and progesterone in CP treated rats**

A single intraperitoneal injection of CP (150 mg/kg) caused a significant decrease in serum estrogen and progesterone (p<0.001) compared to control group. However, administration of ATV at the dose of 10 mg/kg in CP-treated rats significantly restored the levels of estrogen and progesterone in serum, as compared to CP-treated group (p<0.001 and p<0.01, respectively) (Table I).

**Effects of ATV on ovarian histopathological changes in CP-treated rats**

The photomicrographs of ovarian sections in the all groups are shown in Figure 3. In control group, ovary had normal structure in the cortex and medulla (Figure 3-A). Rats treated with ATV showed similar histology to control group (Figure 3-B). Many adverse histological changes were observed in ovary sections of rats treated with CP. They ate including invagination of germinal epithelium along its surface, flattening of germinal epithelium with deeply stained nuclei and losing their arrangements. 

In the ovarian stroma was evident severe acute inflammation, degenerative cells, stromal edema, vacuolization, atresia of the follicles and congestion of blood vessels. Some of the Graafian follicles have appeared with a large antrum and degeneration of zona pellucida and cumulus oophorus. The corpora lutea has occupied a large area in sections (Figure 3-C). Ovary of rats treated daily with ATV for 10 days followed by CP injection exhibited mild degeneration of ovary with less structure changes germinal epithelium cells, mild congestion. Follicles, theca interna and externa were able to preserved their appearance at normal levels (Figure 3-D). Histopathological changes were significantly mitigated in CP+ATV group as compared with CP group. Ovarian injury’s mean scores of all groups are showed in Figure 4. CP-increased ovarian injury score as compared to control group (p<0.001). The Score of ovarian injury in CP treated rats with ATV administration was lower compared to CP group (p<0.01).

**Effect of ATV on immunoreactivity level of caspase-3 in CP-treated rats**

**Figure 5:**
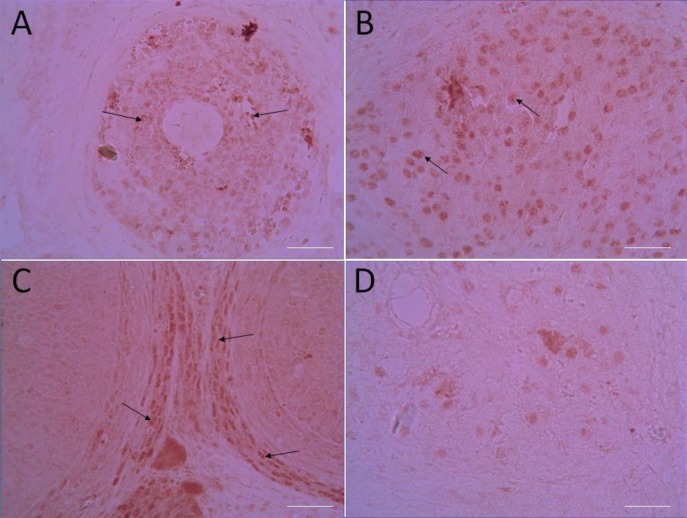
The caspase-3 immunoreactivity in ovarian tissues in CP-induced ovarian injury. CP group: Immunoreactivity (brown color) to caspase-3 was observed in granulosa (A-arrow) and luteal cells (B-arrow), theca externa (C-arrow) in CP treated ovaries as shown in a micrographs. CP+ATV group: moderate immunoreactivity in the ovary gland (D). (Mag. x40). Scale bar = 150 µm

Results of immunohistochemical staining of the ovary are summarized in Figure 5. Sections of ovarian showed no caspase-3 immunoreactivity in the control group. Immunoreactivity level of caspase-3 in the ATV group was similar to control group. Sections of ovarian tissue in CP-treated rats displayed increase immunoreactivity level of caspase-3. Immunoreactivity mainly localized in the granulose (Figure 5.A) and stromal cells, corpus luteum (Figure 5.B) and theca externa (Figure 5.C) of the ovary. While ATV+CP group showed mild immunoreactivity level of caspase-3 in the granulosa and stromal cells and theca externa compared to CP-treated ovary (Figure 5.D). Immunoreactivity of caspase-3 was confirmed by densitometry analysis in rats receiving CP as compared to other groups. 

**Figure 6:**
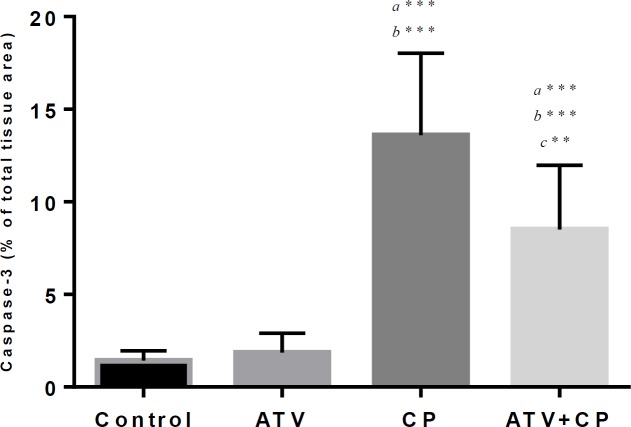
Histogram shows densitometry analysis of immunohistochemical staining for caspase-3. Data were presented as a percentage of total tissue area. Data are presented as Mean ± SD. a vs. the control; b vs. ATV; c vs. CP groups. **; p<0.01, *** and p<0.001.

The histograms of the semi-quantitative analysis of caspase-3 staining are shown in Figure 6. The most intense immunoreactivity of caspase-3 was showed by semi-quantitative analysis in CP treated rats (13.6±4.4.43) compared with control and ATV groups (p<0.001). ATV administration reduced the intensity of immunoreactivity of caspase-3 in CP treated rats (8.51±3.45) (p<0.01). Immunoreactivity level of caspase-3 in the control and ATV groups nearly was to each other (p>0.05).

**Table I:** Effect of ATV on CP-treated rat in serum estrogen and progesterone levels in the all groups

**Groups **	**Control**	**ATV**	**CP**	**ATV+CP**
**Hormone **				
Estrogen (ng/l)	11.6 ± 0.56	11.38 ± 1.56	5.24 ± 1.2 a***^b^***	9.446 ± 1.53 c***
Progesterone (ng/l)	6.39 ± 0.95	6.28 ± 1.1	1.95 ± 0.17 a***^b^***	4.78 ± 0.97 c**

All values are expressed as mean±SD.

ATV: Atorvastatin

CP: Cyclophosphamide

** ; p<0.01,

*** and p<0.001

a significant vs. control

b significant vs. ATV and

c significant vs. CP groups..

ATV: Atorvastatin

CP: Cyclophosphamide

MDA: Malondialdehyde

ROS: Reactive oxygen species

ATV: Atorvastatin

CP: cyclophosphamide

ATV: Atorvastatin

CP: Cyclophosphamide

## Discussion

Chemotherapy drugs, such as CP, due to damage to follicles are extremely toxic for ovarian (5). Previous study showed ATV at low dose have no side effects or toxicity on the ovary (18). In this study, single dose of CP with 150 mg/kg were investigated on ovaries of rats and evaluated the protective effect of ATV against ovarian toxicity induced by CP. We demonstrated that ATV treatment improved estrogen and progesterone levels and decreased MDA, ROS levels, protein carbonyl content and increased the cell viability in CP-treated rat. Also, ATV caused the decrease immunoreactivity level of caspase-3 (apoptosis) and preserved ovarian histological structure. 

CP induces DNA damage in tumor cells and it is benefit for survival of cancer patients, but have side effects on the female reproductive system, such as temporary or permanent amenorrhea, reduced fertility, or infertility (24). Human primordial and antral follicles are sensitive to some drugs. Reduction of the follicles in ovary makes the permanent infertility (25). Phosphoramide mustard, as one of the cytotoxic metabolite of CP, by raising the ROS causes oxidative stress, lipid peroxidation (6) and subsequently leading to granulosa cell apoptosis and increased antral follicle atresia (26). In the present study, MDA and ROS levels, protein carbonyl content and cell viability rate as parameters of oxidative stress, clearly increased in the ovary of CP-treated rat. These changes shown that CP treatment caused oxidative injury to the lipids and proteins in ovary organ. Administration of antioxidant is effective in protecting fertility in women undergoing chemotherapy (5). 

ATV administration in CP-treated rats significantly decreased MDA, PC, ROS levels and significantly increased cell viability rate. These results showed that ATV treatment could protect ovary injury against the side effects of CP. ATV at low dose is well known for its antioxidant and anti-inflammatory properties (10, 11). It is suggested that ATV through antioxidant property may have protective role and improve the antioxidant status of the ovary. In previous studies were demonstrated that ATV administration at two doses of 5 and 10 mg/kg for 30 min before ischemia/reperfusion could have protective effects on ovarian tissue damage. Parlakgumus and colleagues reported ATV (10 mg/kg/day) 7 days before and 7 days after the torsion/detorsion had protective effect on ovarian injury (18). ATV was able to inhibit inflammatory responses and oxidative stress that prevented bleeding and reduced vascular dilatation and edema (27). 

In this study, ATV treatment, at dose 10 mg/kg as pre and post treatment significantly protected rat ovaries against CP-induced toxicity through inhibit lipid and protein peroxidation and maintain cell viability rate. In addition, ATV was used as antioxidant to detect the scavenging activity against ROS induced by CP. Combination therapy ATV with CP revealed that, ROS levels significantly decreased in comparison to CP group. ATV through inhibition the generation of ROS have antioxidant properties (28). Protective effect of atorvastatin has been proven in the intestines (29), brain (30), kidney (15), heart (16), testis (17) and ovary (18) injury against induced ischemia/reperfusion, chemotherapy and irradiation. Chemoprotective and therapeutic effects of ATV has been seen against doxorubicin-induced hepato-renal toxicity (31). Sathyapalan and colleagues showed that ATV in patients with polycystic ovary and hyperandrogenemia reduced inflammation and metabolic parameters after 12 wk (32). 

Inflammation is a physiological process in ovulation, menstruation, and implantation (33), but uncontrolled inflammation has detrimental effects on hormone production and ovulation (34). On the other hand, in the ovary, ROS increase during ovulation and steroidogenesis (35) and detoxification of ROS in oocyte maturation and embryo development is very important (36). ROS with induction lipid peroxidation and production of MDA interfere with ovarian functions (5). Lipid peroxidation in plasma membrane of luteal cells was destroyed gonadotrophin receptors and so decreased steroidogenesis in corpora luteal (37). Regarding hormonal condition, this study showed a significant reduction in estrogen and progesterone after CP administration and is consistent with other studies (6). Saleh showed up in his study that CP induces severe toxicity in the ovary via oxidative stress and inflammation and subsequently decrease E2 and AMH levels in serum (6). 

In this study, ATV could increase level of hormones and preserve ovarian follicles, ovarian function and structure. It seems that taking together both antioxidant and anti-inflammatory properties of ATV in the present study, ATV protected against CP-induced ovarian injury with elevation in serum estrogen and progesterone. Estrogen and progesterone are released by granulosa cells of preantral, small antral follicles and luteal cells. Also, ATV with anti-apoptotic property suppress apoptosis of follicles and luteal cells. In addition, these hormones preserve follicles with inhibition of the initial follicular recruitment from the primordial to the antral and increase hormonal levels (6). Histopathological examination of ovaries pretreated with ATV greatly supported our data as ATV-treated ovary revealed less primordial cell recruitment, more Graafian follicles and corpora lutea at the cortex.

Saleh and co-workers proved a single injection of CP at dose 200 mg/kg destroyed secondary and antral follicles in adult rats but had no side effect on the primary and primordial follicles (6). Also, Lopez and colleagues revealed a single injection of 50 or 300 mg/kg CP creates destruction of the secondary and antral follicles and apoptosis in granulosa cells (38). On the other hand, Parlakgumus and colleagues showed that ATV (10 mg/kg) with increasing AMH and VEGF expression, protected primordial follicles and vascular structures against ovarian injury (18). In the present study, raising the serum levels of estrogen and progesterone by ATV, showed normal growing follicles within ovaries. Histopathological assessment was in accordance with biochemical and immunohistochemical findings.

The overproduction of the free radicals with binding to DNA increase the proapoptotic signals (26). In this signal, cytochrome c from mitochondria enteres the cytosol and then activates caspase-3 and promotes cell death (39). Davis and co-worker showed a single injection of 200 mg/kg CP destroyed secondary and antral follicles (40). CP with induction of apoptosis in granulosa cells (38) and activation of caspase-9 and caspase-3 (41) destroys the secondary and antral follicles. In present study mild immunoreactivity level of caspase-3 was found in ATV+CP group compared to severe immunoreactivity of caspase-3 in CP group. Pre and post-treatment with ATV, as an antioxidant, for 10 days before and after administration of CP decreased apoptosis in growing follicles, stromal cells and luteal corpora. This finding was supported by another study that stated that ATV has anti-apoptotic property (42). The limitation of this study was assessment anti-inflammatory property of ATV.

## Conclusion

Our study concludes that CP could induce histopathological alternation, oxidative stress and apoptosis in the ovary. ATV with anti-oxidative activity and through free radicals scavenging could improve biochemical, hormonal and histopathological parameters. In addition, ovo-protective effect may be attributed to its anti-apoptotic activity that is mediated through suppression immunoreactivity of caspase-3.
